# Effect of Different Injury Morphology of the Endplate on Intervertebral Disc Degeneration: Retrospective Cohort Study

**DOI:** 10.1111/os.14238

**Published:** 2024-10-02

**Authors:** Qiuyu Yu, Kang Chen, Zhongyi Guo, Yaozheng Han, Lintao Su, Changyu Lei, Jun Ma, Hui Kang

**Affiliations:** ^1^ Department of Traumatic Orthopedics, Suizhou Hospital, Hubei University of Medicine Suizhou Hubei China; ^2^ Wuhan University of Science and Technology Wuhan Hubei China; ^3^ General Hospital of Central Theater Command Wuhan Hubei China

**Keywords:** endplate, injury morphology, injury morphology, intervertebral disc degeneration, spinal fractures, thoracolumbar fracture

## Abstract

**Objectives:**

To describe a simplified classification scheme for endplate injury morphology based on 3D CT and to examine possible associations between endplate injury morphology and vertebral space and other variables such as type of fracture and disc degeneration in a group of patients with thoracolumbar fractures.

**Methods:**

This study was a retrospective cohort study. We collected patients with thoracolumbar fractures admitted from January 2015 to August 2020 and divided them into three groups based on the morphology of endplate injury (45 cases of mild endplate injury, 54 cases of moderate endplate injury, and 42 cases of severe endplate injury, SEI). Data of vertebral body and intervertebral space height and angle, the Pfirrmann grade, endplate healing morphology were collected during preoperative, postoperative, and long‐term follow‐up of patients in each group. One‐way analysis of variance (ANOVA), chi‐squared test, and repeated measurement ANOVA were used to compare and analyze the influence of endplate injury morphology on patient prognosis.

**Results:**

Most moderate injuries to the endplate (fissure‐type injury) and severe injuries (irregular depression‐type injury, Schmorl's node‐type injury) resulted in significant disc degeneration in the long‐term transition. This study also showed significant differences in the height of the anterior margin of the injured spine and the intervertebral space height index during this process.

**Conclusions:**

The current study suggests that although the region of injury in endplate fissure‐type injury is small preoperatively, it may be a major factor in leading to severe disc degeneration, loss of intervertebral height, and Cobb angle in the long term. The results of our study therefore may allow surgeons to predict the prognosis of patients with thoracolumbar fractures and guide their treatment.

## Graphical abstract

We propose a simplified classification for the morphology characteristics of endplate injury based on 3D CT: mild, moderate, and severe. Our results indicate that moderate and severe endplate injury cause severe disc degeneration and long‐term loss of intervertebral height and Cobb angle. This may allow surgeons to predict patient prognosis and guide treatment for thoracolumbar fractures.

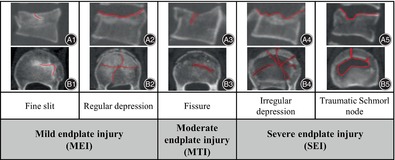

## Introduction

The thoracolumbar region is recognized as one of the most common areas where spinal fracture takes place.^1^ Intervertebral disc (IVD) injury, as the most common concomitant injury to thoracolumbar segment fractures, has attracted a great deal of attention in recent years. Some patients with thoracolumbar fractures suffer from complications such as loss of intervertebral space height, kyphotic deformity, and even long‐term intractable lower back pain due to severe disc degeneration after the removal of internal fixation, which greatly affects the quality of life of patients.^2^, ^3^, ^4^ Therefore, it seems to be insufficient to assess the long‐term prognosis of those patients only by their vertebral height, neurological symptoms, and segmental stability after internal fixation.^4^, ^5^, ^6^ Recently, a large amount of literature has suggested that the endplate plays an important role in material transport, stress dispersion, and immune protection in the spine, and any factor that interferes with these mechanisms may be a potential risk for Intervertebral disc degeneration (IDD).^4^, ^7^, ^8^, ^9^

An accepted standard for the assessment of endplate injuries lacks around the world. Brayda et al.^10^ explored the relationship between endplate injury morphology and lower back pain by dividing endplate injury morphology into five groups on MRI and believe that “wavy/irregular” and “Schmorl's node” are closely associated with rapid IDD. However, the endplate injury classification in their study was too complex, a more comprehensive assessment of endplate injury by 3D CT imaging was not performed, and the differences in prognosis between the various endplate injury morphologies were not reflected in the results. Therefore, the relationship between the various injury morphologies of the endplate and IDD and its prognostic profile is still not well understood.

In this paper, we attempted to reintegrate endplate injury morphology classification in patients with thoracolumbar fractures and hypothesized that different classification of endplate injury morphology would have different effects on the course of IDD. The purpose of this study was (i) to simplify the morphological classification of endplate injury for clinical application; (ii) to investigate the endplate injury morphology relationships with long‐term prognosis in patients with thoracolumbar fractures; and (iii) to provide references for clinical prognosis assessment.

## Materials and Methods

This was a retrospective cohort study. Patients admitted to the Department of Orthopedics, General Hospital of Central Theater, from January 2015 to August 2020, with thoracolumbar spine fracture were followed up from 12 to 26 months, and indices related to thoracolumbar fracture were collected and measured. Approval from the Ethics Committee of the General Hospital of the Central Theater Command was obtained prior to November 3, 2021 (Ethics No. [2021] 059–01) and did not violate the Declaration of Helsinki. All the patients had consented to the use of their data.

### Patient Inclusion and Exclusion

Inclusion criteria were as follows: (1) The patients are young and middle aged (from 18 to 55 years old); (2) fracture of a single vertebral body in T11‐L4 and the adjacent vertebrae of the injured vertebra was intact; (3) they were treated with pedicle screw internal fixation; and (4) 3D CT images showed that the superior endplate was injured in the injured vertebrae, but the inferior endplate was intact.

Exclusion criteria were as follows: (1) history of previous surgery, infection, tumor, and so forth; (2) congenital malformation, previous history of lumbar spine trauma^11^, ^12^; (3) severe loss of intervertebral height (Sander Grade 3 or higher), severe fracture‐dislocation; (4) presence of neurological disease, muscle tissue disease that prevents long‐term functional exercise; and (5) incomplete imaging data.

### Classification for the Endplate Injury

**FIGURE 1:**
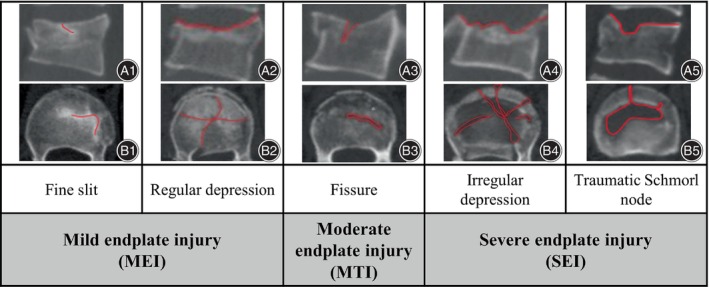
Endplate classification. (A) CT 3D lateral view. (B) CT 3D axial view. (Red line) the fracture line. Group 1 includes fine slit and regular depression. Group 2 includes fissure. Group 3 includes irregular depression and traumatic Schmorl's node. “Fine slit”: The vertebral body is intact or slightly compressed, the compressed vertebral body height <20%, with normal intervertebral disc (IVD) height.^13^ A linear injury can be seen in the injured endplate, and no significant soft tissue invasion of the vertebral body was observed in 3D CT. “Fissure”: The vertebral body is intact or slightly compressed, the compressed vertebral body height <20%, with normal IVD height. A fissure‐type injury can be seen in the injured endplate, and a small portion of soft tissue invasion can be seen in 3D CT. “Regular depression”: The vertebral body collapses obviously, the compressed vertebral body height >20%, with mild loss of IVD height. The depressed endplate is regular, and there is no significant soft tissue invasion of the vertebral body in 3D CT. “Irregular depression”: The vertebral body collapses obviously, the compressed vertebral body height >20%, with mild loss of IVD height. The depressed endplate is irregular, and much soft tissue invades of the vertebral body, which can be seen on 3D CT. “Traumatic Schmorl's‐node”: The depressed endplate has a shape similar to the Schmorl's‐node and much soft tissue invades the vertebral body, with significant loss of IVD height.

Given that the nature of the endplate is cartilage, we believe that its injury morphology is more readily identifiable on 3D CT. We reorganized the endplate injuries according to their characteristics such as depth and area observed in 3D CT based on the research by Brayda et al.^10^ The injuries were classified into five categories: fine slit, fissures, regular depression, irregular depression, and traumatic Schmorl's node, which facilitate easier differentiation of them in clinical practice. Taking into account their potential impact on patients' long‐term prognosis, we simplified and merged these categories into three groups. In our study, these patients were classified into group MEI (mild endplate injury): fine slits and regular depressions; group MTI (moderate endplate injury): fissure; and group SEI (severe endplate injury): irregular depressions and traumatic Schmorl's node (Figure 1).

### Classification of Endplate Healing Morphology

**FIGURE 2:**
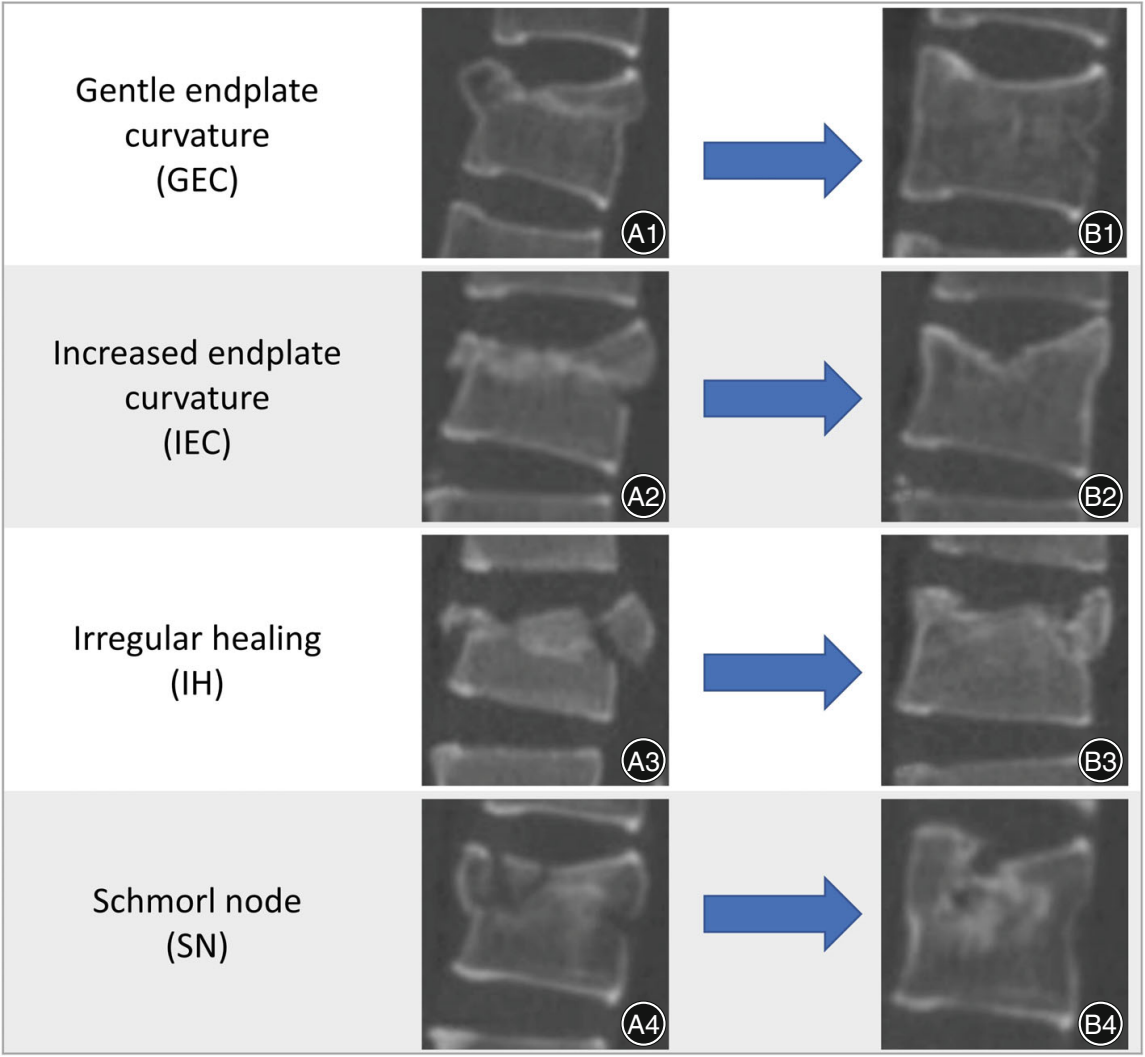
Endplate healing morphology. (A) Preoperative CT 3D lateral view. (B) CT 3D lateral view after the removal of internal fixation. Gentle endplate curvature (GEC): endplate depression repair with a significant increase in depression angle and no significant loss of intervertebral disc (IVD) height. Increased endplate curvature (IEC): endplate depression repair with a significant reduction in depression angle and no significant loss of IVD height. Irregular healing (IH): endplate depression signal irregularity with localized elevation or depression, mild loss of IVD height. Schmorl's node (SN): endplate depression similar to Schmorl's node with a significant loss of IVD height.

In the analysis and study of the morphology of endplate healing, we refer to the research methods of Su et al.^4^ (Figure 2).

Data collection and evaluation were performed by an attending physician and an experienced deputy chief surgeon in spine surgery. Two months after the completion of the first evaluation, the spinal associate chief surgeon measured all the data again in the same way on his independent workstation, and the final result was determined in consultation with the two surgeons as to whether the average value was used as the final result. Intra‐ and inter‐rater reliability of the measurements were estimated.

### Surgical Procedures

All patients underwent comprehensive preoperative imaging of the thoracolumbar spine, encompassing frontal and lateral radiographs, 3D CT and MRI of the thoracic/lumbar spine. The surgical approach, either open reduction and internal fixation utilizing pedicle screws or percutaneous pedicle screw fixation, was determined based on factors such as the severity of vertebral body compression, the presence of posterior longitudinal ligament injury, and the occurrence of neurological manifestations. The surgical internal fixation stabilization system employed in these procedures was sourced from Shanghai Sanyou Company in China. All surgeries were executed under general anesthesia, adhering to rigorous surgical protocols. Following hospital discharge, patients were instructed to engage in active ambulation, facilitated by the use of a thoracolumbar brace for protection. At the three‐month postoperative mark, patients were advised by their physicians to discontinue the use of the thoracolumbar brace, contingent upon the assessment of bone healing as evidenced by 3D CT. The mean duration for the removal of pedicle screws and connecting rods was 18 months, with a range spanning from 10 to 24 months.^14^

### Data Collection, Measurements, and Definition

**FIGURE 3:**
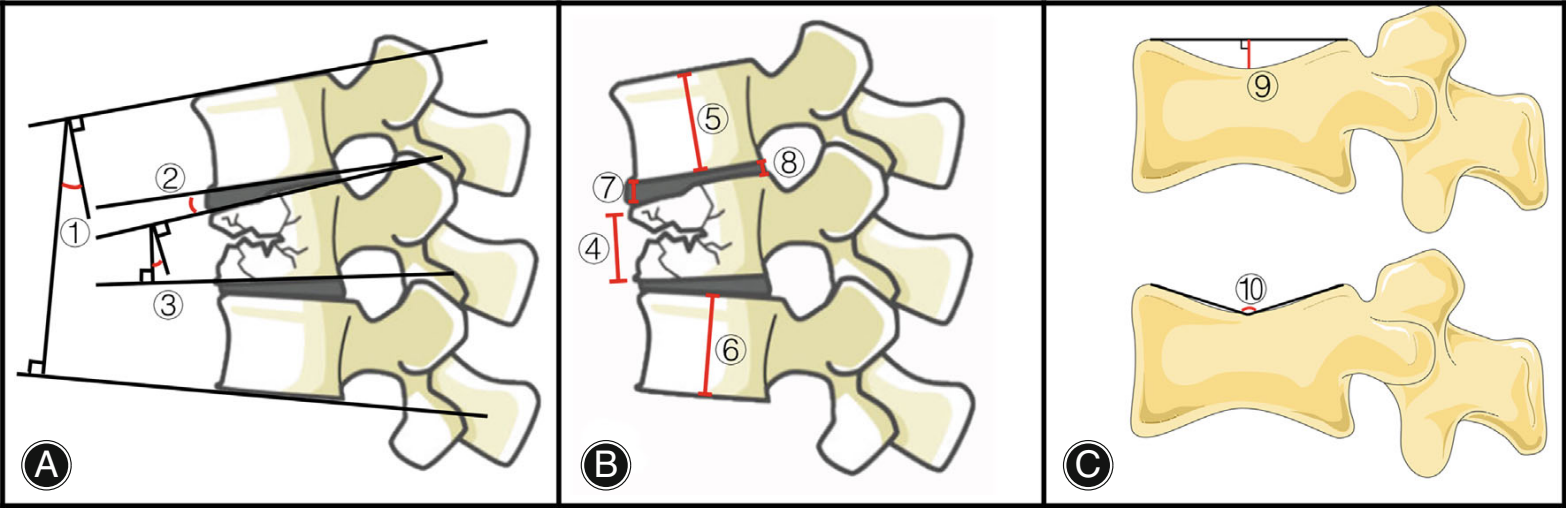
Imaging measurements. (A) Angle measurement in CT sagittal images. (B) Length measurement in CT sagittal images. (C) Measurement of endplate depression in CT sagittal images. ① Sagittal Cobb angle (SCA). ② Intervertebral Cobb angle (ICA). ③ Vertebral wedge angle (VWA). ④ Anterior height of the injured vertebra (AH). ⑤ Superior vertebral body height (SVH) of the injured vertebrae. ⑥ Inferior vertebral body height of the injured vertebrae (IVH). ⑦ Anterior heights of the intervertebral space (AHI). ⑧ Posterior heights of the intervertebral space (PHI). ⑨ Depth of the endplate (DE). ⑩ Endplate depression angle (EDA).

The preoperative Sander's classification^15^ and Pfirrmann's classification^16^ of the injured vertebral supraspinal disc were assessed by MRI, combined with CT 3D images to evaluate injured vertebrae according to Denis classification.^17^ To avoid interference with Pfirrmann's classification due to disc edema, we determined the degeneration of the target disc by analyzing the degeneration of adjacent discs above and below the target disc. The imaging measurements included Sagittal Cobb angle (SCA), anterior height (AH) of the injured vertebra, vertebral wedge angle (VWA), superior vertebral body height (SVH) of the injured vertebrae, inferior vertebral body height of the injured vertebrae (IVH), anterior heights of the intervertebral space (AHI), posterior heights of the intervertebral space (PHI), intervertebral Cobb angle (ICA), endplate depression angle (EDA), and depth of the endplate (DE) as reported before^13^, ^18^, ^19^ (Figure 3). Calculation of the intervertebral disc height index (DHI) based on the results of intervertebral space, superior, and inferior vertebral body height measurements.^20^

$$\mathrm{DHI}=\frac{\mathrm{AHI}+\mathrm{PHI}}{\mathrm{SVH}+\mathrm{IVH}}.$$

To reduce the human error caused by the measurement, the values in this calculation are expressed as the ratio of the measured value to the average of the superior and inferior vertebral body heights of the injured vertebrae measured at that time (except for the angular values).

For example:

$${\text{Ratio}}_{\left({\mathrm{AH}}_{\mathrm{pre}}\right)}=\frac{2*{\mathrm{AH}}_{\mathrm{pre}}}{{\mathrm{SVH}}_{\mathrm{pre}}+{\mathrm{IVH}}_{\mathrm{pre}}}.$$

We evaluated the agreement between the two physicians on the morphological classification system of endplate injuries by the Fleiss Kappa statistic and the agreement of the continuous variables measured by the two physicians in this study at three time points (pre‐op, post‐op, and F/U) by the intraclass correlation coefficient (ICC).

### Statistical Analysis

IBM SPSS 25.0 (IBM Corporation, Armonk, NY) was used for all statistical analysis. Measures were expressed as mean ± standard deviation or number of cases (N%). One‐way analysis of variance (ANOVA) was used for the comparison of continuous variables between groups, and chi‐squared test or Fisher's exact test was used for the comparison of constituent ratio of categorical variables and rank variables. Repeated measurement ANOVA was used to study the morphology of endplate injury and the changes of IVD and the healing trend in its related indexes in the long‐term injured vertebrae. Post hoc Bonferroni correction to adjust for multiple comparisons was utilized (the *p*‐value cutoff for Bonferroni correction = 0.05/3).

## Results

### Patient Characteristics

**FIGURE 4:**
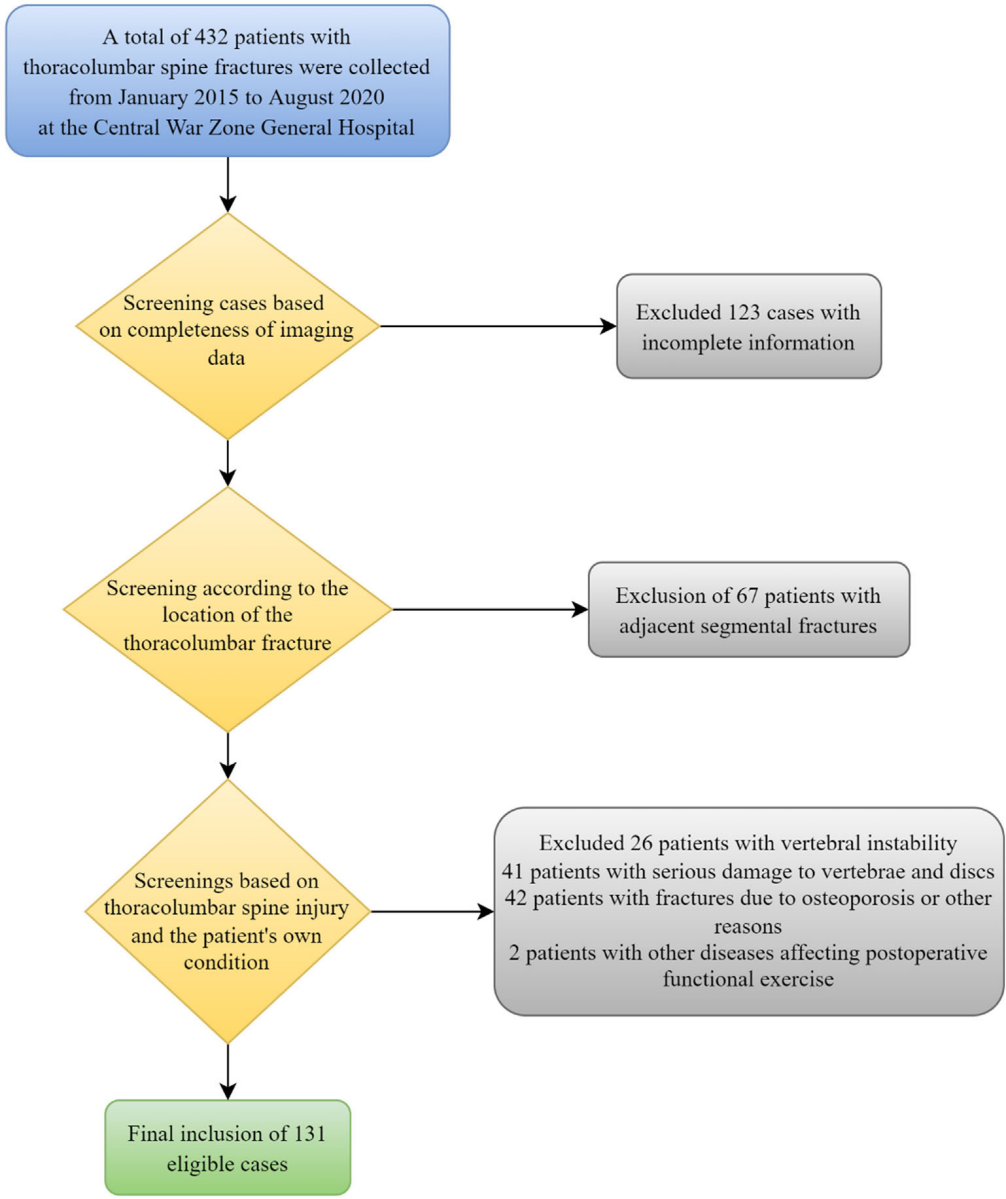
Case screening strategy.

A total of 432 patients were involved in this study, and 131 patients (83 males and 48 females) with thoracolumbar fractures were finally included according to the inclusion and exclusion criteria. Of these, 10 cases (7 males and 3 females) were repeatedly included because of discontinuous vertebral fractures (Figure 4). Based on the results of Kappa statistical of endplate injury morphology (Kappa ≈ 0.84, *p* < 0.001), pre‐op (ICC, 0.855–0.988), post‐op (ICC, 0.822–0.981), and F/U (ICC, 0.870–0.990), we concluded that there was a strong agreement between two independent observers.

Three groups of demographic data were collected and compared, including age, sex, cause of injury, fracture site, and neurological status. After statistical analysis, there were significant differences between the three groups of patients in terms of TLICS score, fracture level, Denis typing, degree of bone edema, and Sander grading (*p* < 0.05) (Table 1).

**TABLE 1:** Comparison between demographic characteristics in three groups.

Characteristics	MEI (*n* = 45)	MTI (*n* = 54)	SEI (*n* = 42)	*p*‐value
Age (years)	39.76 (±14.935)	39.98 (±12.354)	40.60 (±12.939)	>0.05
Sex	26 (57.8%)	38 (70.4%)	26 (61.9%)	>0.05
TLICS score	5.40 (±0.809)	5.04 (±1.081)	5.79 (±0.968)	**<0.001**
Mechanism of fracture				>0.05
Traffic accident	9 (20.0%)	10 (18.5%)	4 (9.5%)	
Fall from height	15 (33.3%)	24 (44.4%)	27 (64.3%)	
Fall from floor	19 (42.2%)	17 (31.5%)	10 (23.8%)	
Other	2 (4.4%)	3 (5.6%)	1 (2.4%)	
Fracture level				**<0.05**
T11	2 (4.4%)	0 (0.0%)	0 (0.0%)	
T12	10 (22.2%)	10 (22.2%)	6 (14.3%)	
L1	24 (53.3%)	23 (42.6%)	60 (31.0%)	
L2	7 (15.6%)	11 (20.4%)	15 (35.7%)	
L3	2 (4.4%)	7 (13.0%)	2 (4.8%)	
L4	0 (0.0%)	3 (5.6%)	5 (11.9%)	
Denis typing				**<0.001**
Denis I B	27 (60.0%)	39 (72.2%)	14 (33.3%)	
Denis I D	9 (20.0%)	0 (0.0%)	0 (0.0%)	
Denis II B	9 (20.0%)	15 (27.8%)	28 (66.7%)	
Bone edema level				**<0.001**
<1/3	26 (57.8%)	21 (38.9%)	3 (6.7%)	
1/3–2/3	16 (35.6%)	19 (35.2%)	14 (25.9%)	
>2/3	3 (6.7%)	17 (40.5%)	17 (40.5%)	
ASIA impairment scale				>0.05
B	1 (2.2%)	2 (3.7%)	2 (4.8%)	
C	0 (0.0%)	0 (0.0%)	2 (4.8%)	
D	4 (8.9%)	4 (7.4%)	9 (21.4%)	
E	40 (88.9%)	48 (88.9%)	29 (69.0%)	
Sander's classification				**<0.001**
Grade 0	15 (33.3%)	5 (9.3%)	0 (0.0%)	
Grade 1	23 (51.1%)	29 (53.7%)	15 (35.7%)	
Grade 2	7 (15.6%)	20 (37.0%)	27 (64.3%)	
Pfirrmann's classification				>0.05
Grade 1	27 (60.0%)	30 (55.6%)	17 (40.5%)	
Grade 2	17 (37.8%)	19 (35.2%)	21 (50.0%)	
Grade 3	1 (2.2%)	5 (9.3%)	4 (9.5%)	

*Note*: Values are presented as mean ± SD or *N*%. The *p*‐value of age and TLICS score was calculated by one‐way ANOVA; others are calculated by chi‐squared test or Fisher's exact test.

### Analysis of the Influence of the Shape of Endplate Injury on the Grade of IDD

**FIGURE 5:**
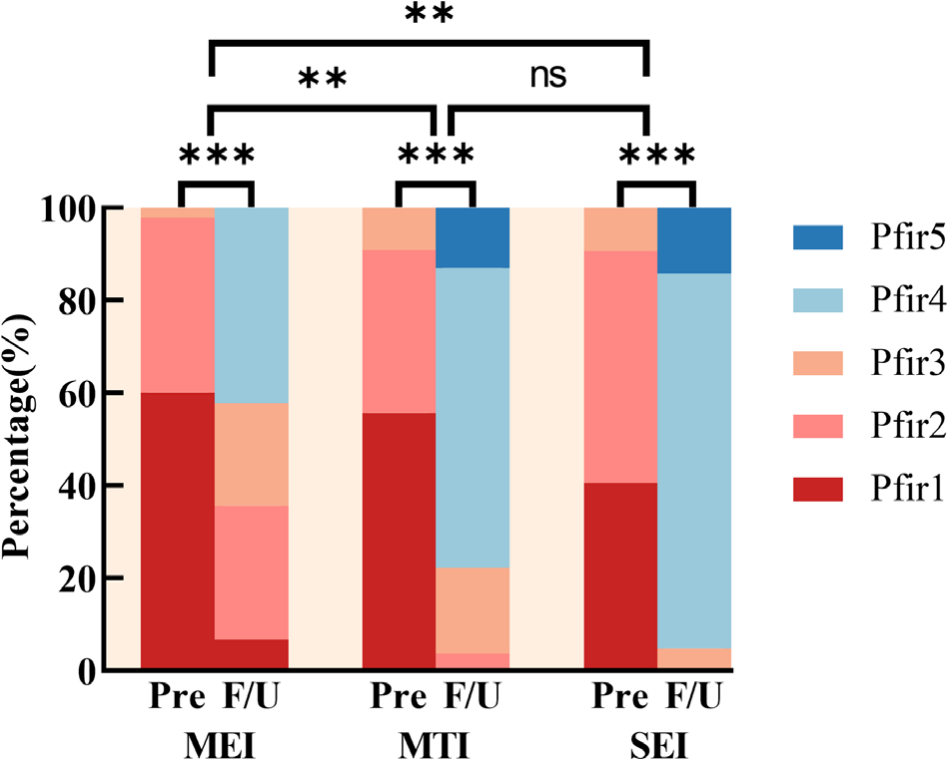
Changes in Pfirrmann's classification of disc degeneration in the upper intervertebral disc (IVD) of the injured vertebrae. The intergroup comparison in the figure was to compare and analyze the distribution of Pfirrmann's classification during the long‐term follow‐up of the three groups after surgery. *p < 0.05; **p < 0.01; ***p < 0.001.

After different degrees of endplate injury, the long‐term outcome of the MTI group and the SEI group showed an obvious accelerated trend in IDD. As can be shown in Figure 5 and Table 2, Pfirrmann's grade of the upper IVD of the injured vertebrae in the three groups of patients included in this study was mainly 1–2 (Pfir1 MEI_Pre_ + Pfir2 MEI _Pre_ = 44, 97.8%; Pfir1 MTI_Pre_ + Pfir2MTI_Pre_ = 49, 90.8%; Pfir1 SEI_Pre_ + Pfir2 SEI_Pre_ = 38, 90.5%). After internal fixation treatment, all the patients in the three groups showed varying degrees of IDD. After 10–26 months of treatment, the proportion of people with Pfirrmann's classification of 4–5 in the MTI group (Pfir4 MTI_pre_ + Pfir5 MTI_pre_ = 0, 0.0% vs. Pfir4 MTI_F/U_+ Pfir5MTI_F/U_ = 42, 77.8%) and the SEI group (Pfir4 SEI_pre_ + Pfir5 SEI_pre_ = 0, 0.0% vs. Pfir4 SEI_F/U_ + Pfir5 SEI_F/U_ = 40, 95.3%) was significantly more than that in the MEI group (Pfir4 MEI_pre_ + Pfir5 MEI_pre_ = 0, 0.0% vs. Pfir4 MEI_F/U_+ Pfir5 MEI_F/U_ = 19, 42.2%), showing some similarity.

**TABLE 2:** Changes in Pfirrmann's classification of disc degeneration in the upper intervertebral disc (IVD) of the injured vertebrae.

Pfirrmann's classification	MEI*		MTI*		SEI*	
	Pre	F/U^a^	Pre	F/U^b^	Pre	F/U^b^
Pfir1	27 (60%)	3 (6.7%)	30 (55.6%)	0 (0%)	17 (40.5%)	0 (0.0%)
Pfir2	17 (37.8%)	13 (28.9%)	19 (35.2%)	2 (3.7%)	21 (50.0%)	0 (0.0%)
Pfir3	1 (2.2%)	10 (22.2%)	5 (9.3%)	10 (18.5%)	4 (9.5%)	2 (4.8%)
Pfir4	0 (0%)	19 (42.2%)	0 (0%)	35 (64.8%)	0 (0%)	34 (81.0%)
Pfir5	0 (0%)	0 (0%)	0 (0%)	7 (13.0%)	0 (0%)	6 (14.3%)

*Note*: The *p*‐value was calculated by chi‐squared test or Fisher's exact test.

* Pre versus F/U *p* < 0.05; (a, b) groups that do not share the same letter are significantly different from each other (*p* < 0.05).

### Analysis of the Influence of Morphology of Endplate Injury on the Height of Intervertebral Space

**FIGURE 6:**
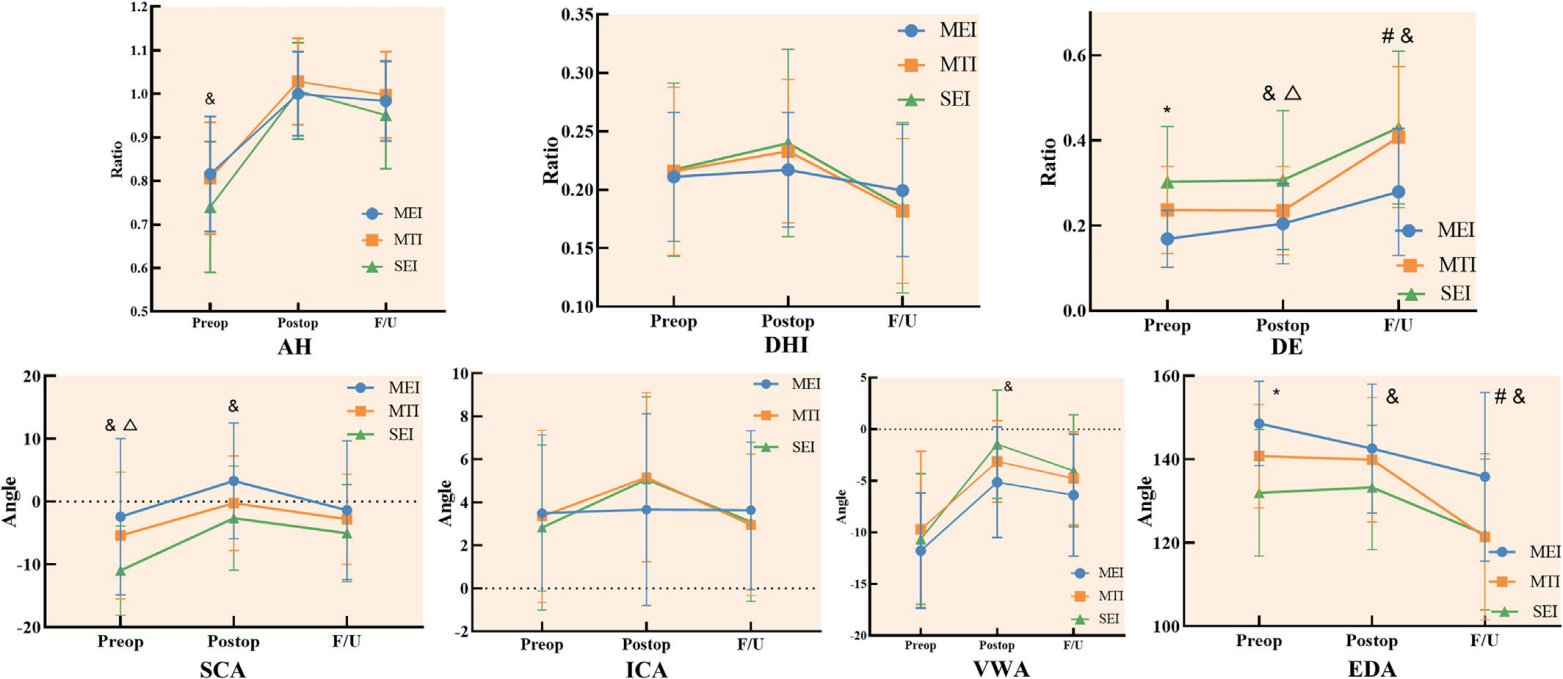
Trend in indexes of height and angle healing in three groups. AH, anterior height of the injured vertebra; DHI, intervertebral disc height index; DE, depth of the endplate; EDA, endplate depression angle; ICA, intervertebral Cobb angle; SCA, sagittal Cobb angle; VWA, vertebral wedge angle. The *p*‐value was calculated by one‐way ANOVA. #, MEI versus MTI *p* < 0.05; &, MEI versus SEI *p* < 0.05; △, MTI versus SEI *p* < 0.05; *, MTI versus SEI *p* < 0.05.

Statistical analysis showed that the healing trend in the MEI group was significantly different from that of the MTI group in the three indexes of height. In preoperative AH, the SEI group (*n* = 42, AH SEI_Pre_ = 0.740 ± 0.021) was relatively lower than the MEI group (*n* = 45, AH MEI_Pre_ = 0.816 ± 0.020) and the MTI group (*n* = 54, AH MTI_Pre_ = 0.806 ± 0.019) (^&^ *p* < 0.05), but the same level could be achieved in all three groups after surgical treatment. In DHI, there was no significant difference among the three groups at the three time points of preoperative, postoperative, and long‐term follow‐up, but the healing trend in the SEI group was significantly different from that of the MEI and MTI groups (*p* < 0.05), and the healing trend in the MEI group was relatively flat. There were significant differences in preoperative DE among the three groups (DE MEI Pre = 0.169 ± 0.015, DE MTI Pre = 0.237 ± 0.014, DE SEI Pre = 0.303 ± 0.016; *p < 0.05). After surgical treatment, the endplate depression in the MTI group was significantly repaired, which was similar to that in the MEI group. However, in the long‐term follow‐up, the endplate depression of the MTI group was significantly deepened again, similar to that of the SEI group. The prognosis of the MEI group was significantly better, and the healing trend was significantly different from that of the MTI group (Figure 6, Table 3).

**TABLE 3:** Trend in indexes of height healing in three groups.

Indexes of height	MEI	MTI	SEI
AH^#&△^			
Preop	0.816 (±0.020)	0.806 (±0.019)	0.740 (±0.021)
Postop	1.000 (±0.015)	1.028 (±0.014)	1.007 (±0.016)
F/U	0.984 (±0.016)	0.998 (±0.014)	0.951 (±0.016)
DHI^#&^			
Preop	0.211 (±0.055)	0.216 (±0.072)	0.217 (±0.074)
Postop	0.217 (±0.048)	0.233 (±0.061)	0.240 (±0.080)
F/U	0.199 (±0.057)	0.182 (±0.062)	0.185 (±0.073)
DE^#^			
Preop	0.169 (±0.015)	0.237 (±0.014)	0.303 (±0.016)
Postop	0.205 (±0.018)	0.235 (±0.017)	0.307 (±0.019)
F/U	0.279 (±0.025)	0.408 (±0.022)	0.430 (±0.025)

*Note*: *p*, Healing trends in the three groups of patients. The *p*‐value was calculated by repeated measurement ANOVA. #, MEI versus MTI *p* < 0.05; &, MEI versus SEI *p* < 0.05; △, MTI versus SEI *p* < 0.05.

Among the Angle indexes, the SCA of the MEI group was the largest (−2.43 ± 12.461) before operation, which meant that some patients in this group still retained the lordosis of the vertebral body, while most patients in the SEI group (−11.00 ± 7.129) had severe thoracolumbar kyphosis before operation. After surgical treatment, the kyphotic deformity was significantly repaired in all three groups, and there was no significant difference in long‐term follow‐up. However, there was a significant difference in the healing trend between the MEI and SEI groups. In this study, there was no significant difference in the healing trend in ICA and VWA among the three groups. In EDA, there were significant differences among the three groups before operation, and the MEI group had the smallest depression (148.56 ± 10.10). After surgical repair, the depression of endplate in the three groups was not significantly improved. In the long‐term, the MEI group (135.80 ± 20.15) showed the least degree of endplate depression, which was significantly different from the MTI group (121.43 ± 19.91) and the SEI group (122.00 ± 18.07). There was also a statistically significant difference in the healing trend between the MEI and MTI groups (Table 4).

**TABLE 4:** Trend in indexes of angle healing in three groups

Indexes of angle	MEI	MTI	SEI
VWA			
Preop	−11.76 (±5.605)	−9.70 (±7.568)	−10.64 (±6.328)
Postop	−5.13 (±5.354)	−3.11 (±3.946)	−1.45 (±5.255)
F/U	−6.38 (±5.910)	−4.76 (±4.505)	−4.02 (±5.426)
SCA^&^			
Preop	−2.43 (±12.461)	−5.41 (±10.100)	−11.00 (±7.129)
Postop	3.29 (±9.208)	−0.26 (±7.516)	−2.64 (±8.277)
F/U	−1.40 (±11.048)	−2.83 (±7.189)	−5.04 (±7.737)
ICA			
Preop	3.51 (±3.628)	3.35 (±4.001)	2.83 (±3.838)
Postop	3.67 (±4.457)	5.17 (±3.932)	5.07 (±3.841)
F/U	3.64 (±3.694)	2.96 (±3.291)	3.10 (±3.701)
EDA^#^			
Preop	148.56 (±10.10)	140.76 (±12.38)	131.98 (±15.16)
Postop	142.58 (±15.47)	139.93 (±14.90)	133.26 (±14.87)
F/U	135.80 (±20.15)	121.43 (±19.91)	122.00 (±18.07)

*Note*: *p*: Healing trends in the three groups of patients. The *p*‐value was calculated by repeated measurement ANOVA. #, MEI versus MTI *p* < 0.05; &, MEI versus SEI *p* < 0.05; △, MTI versus SEI < 0.05.

### Analysis of the Relationship between Endplate Injury Morphology and Healing Morphology

**FIGURE 7:**
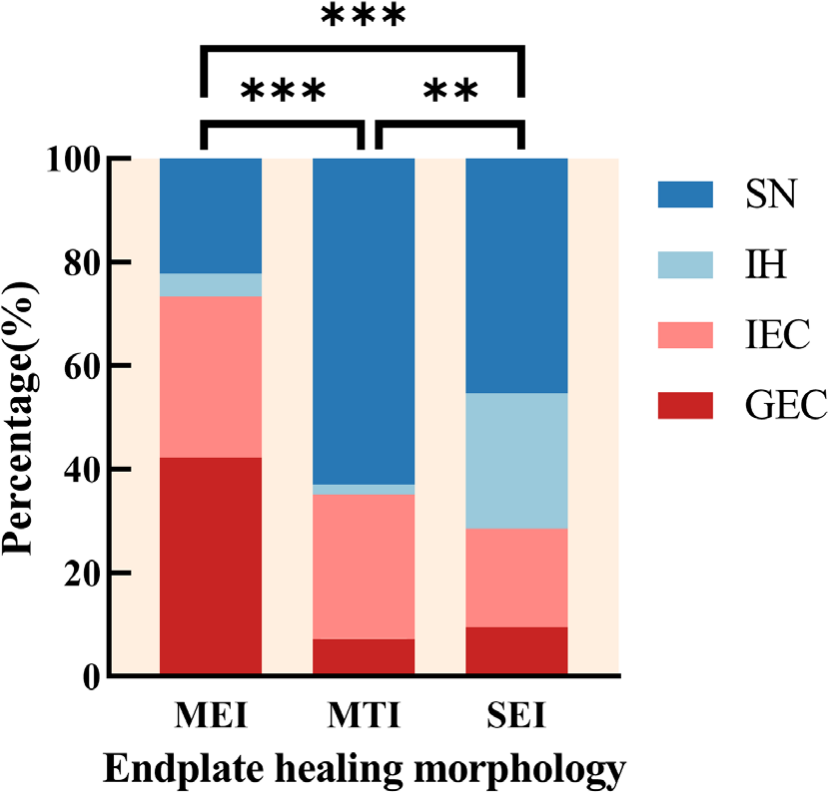
Distribution of the endplate healing morphology. * *p* < 0.05; **p < 0.01; ***p < 0.001.

According to Figure 7 and Table 5, the long‐time healing morphology of the endplate in group MEI patients was mainly GEC and IEC, with GEC having the largest percentage (GEC MEI = 19, 42.2%). The healing morphology of the endplate in the MTI group of patients in the long term was dominated by IEC and SN, which was the group with the largest proportion of conversion to SN among the three groups (SN MTI = 34, 63.0%). IH and SN were the predominant endplate healing patterns in the SEI group, with SN being the most common and the group with the largest proportion (IVH SEI + SN SEI = 31, 71.4%) of endplates converted to unstable patterns (IVH and SN) in the long term.^4^ The incidence of different healing morphology of the endplates was statistically different among the three groups of patients (*p* < 0.01).

**TABLE 5:** Distribution of the endplate healing morphology.

Endplate healing morphology	MEI^a^	MTI^b^	SEI^c^
GEC	19 (42.2%)	4 (7.4%)	4 (9.5%)
ICE	14 (31.1%)	15 (27.8%)	8 (19.0%)
IH	2 (4.4%)	1 (1.9%)	11 (26.2%)
SN	10 (22.2%)	34 (63.0%)	19 (45.2%)

*Note*: a–c, groups that do not share the same letter are significantly different from each other (*p* < 0.05). The *p*‐value was calculated by chi‐squared test.

## Discussion

In this study, a retrospective cohort analysis was conducted to investigate the correlation between the morphology of endplate injury and long‐term IDD in patients with thoracolumbar fractures. We reclassified the patients based on the prominent morphological features of their endplate injuries, referencing previous research.^10^, ^21^, ^22^ This revised endplate injury classification system proves to be simpler, more practical, and demonstrates a high degree of assessment consistency. The findings reveal a strong association between the morphology of the endplate injury and the long‐term prognosis of patients with thoracolumbar fractures. Moderate and SEIs are found to result in poor prognosis for patients, including the loss of IVD height and kyphotic deformity during long‐term transition. This classification system can aid clinicians in assessing the prognosis of thoracolumbar fracture patients, enabling them to devise more tailored and effective treatment plans for each individual.

### Morphological Classification and Prognosis of Endplate Injury

IDD is caused by a variety of factors, and endplate injury that induces disc nutritional impairment, abnormal stress, immune and inflammatory responses is the main cause of disc degeneration.^23^, ^24^, ^25^, ^26^, ^27^ Wang et al.^28^ found that there was a strong association between degeneration of the disc and endplate damage after observing 76 human disc endplate specimens. This is consistent with our research results. Zehra et al.^7^ concluded that the size of endplate injury was an important decisive factor in the degree of IDD by calculating the area of endplate defect. However, it is not realistic for medical workers to accurately measure the area of each patient's endplate defect like Zehra in clinical practice. We believe that a better understanding of the impact of endplate injury morphology on clinical prognosis will effectively improve the efficiency of medical personnel in assessing patients with endplate injury.

In the study of the relationship between endplate healing and IDD by Su et al.,^4^ it was considered that irregular healing‐type and traumatic Schmorl node‐type were closely related to accelerated degeneration of IVD. In this study, we proved the relationship between endplate injury and long‐term IDD. In addition to this, we also analyzed the relationship between endplate injury morphology and long‐term healing morphology which was consistent with previous studies.^7^, ^29^, ^30^ From the results, endplate with fine slit‐type injury or regular depression‐type injury tended to have a benign recovery outcome such as gentle endplate curvature (GEC) or increased endplate curvature in the long term, whereas fissure‐type injury had a strong causal relationship with long‐term Schmorl's node‐type healing. In the long‐term transition, a greater proportion of patients with thoracolumbar fractures with irregularly depression‐type injuries or Schmorl's node‐injuries had poor endplate healing morphology (irregular depression and Schmorl's node).^4^

### The Long‐Term Prognosis of IVD Is Related to the Morphology of Endplate Injury

In past studies, Brayda et al.^10^ have demonstrated the relationship between endplate injury morphologies and disc degeneration and lower back pain. However, in their study, the relationship between many endplate injury morphologies was not clearly described due to the overly complex endplate typing. We believe that it is important for physicians not only to understand which injury morphology predisposes to disc degeneration and its prognosis but also to grade it by prognostic outcome in clinical practice. We found that patients with fine slit‐type or regular depression‐type injury of endplate did not show more significant degeneration of the disc in the distal phase, and no significant loss of IVD height or Cobb angle was seen. Therefore, even though the extent of damage to the endplate is large in regular depression‐type of the endplate, the degree of damage is mild and a good prognosis can still be achieved. We believe that although regular depression changes the stress environment of the endplate and IVD to some extent, it does not excessively expose IVD to the blood environment and reduces the effect of inflammatory reaction and immune response on IDD.^31^ In this study, fissured endplate injury had a more significant impact on IDD, potentially due to the increased exposure of IVD to blood through the endplate with Fissure‐type injury leading to inflammation and immune responses within the intervertebral disc.^26^, ^27^ In the long‐term follow‐up, the fissure‐type injury of endplate showed a significant loss of the IVD height and rapid IDD, compared with the fine slit‐type injury or regular depression‐type injury.

On the contrary, although most preoperative and postoperative indices are better in patients with endplate fissure‐type injury than in patients with irregular depression or Schmorl's nodule injury, after long‐term healing and repair, both DHI and ICA of endplate fissure‐type injuries approached those indices of which patients with irregular depression‐type or Schmorl's node‐type injuries. In endplate fissure‐type injuries, a small amount of disc tissue is embedded in the endplate, which not only alters the stress environment of the endplate but also triggers a local inflammatory response that leads to increased peripheral cell death and collapse of the underlying bone trabeculae.^25^, ^32^, ^33^ Finally, the discs that protrude into the endplate gradually calcify and thicken, forming traumatic Schmorl's node.^24^, ^34^ Although fissure‐type injuries are less severe than irregular depression‐type injuries and Schmorl's node‐type injuries, the long‐term follow‐up results are similar to them. These facts show that endplate fissure‐type injuries can significantly promote upper disc degeneration, with significant differences in clinical prognosis compared with fine slit‐type injuries.

In this study, patients with irregular depression injury and Schmorl's nodule injury had the most SEI of the three groups and achieved the worst long‐term healing. However, postoperative improvement rates were greatest in AH, VWA, and DHI among the three groups. Severe endplate injuries are often accompanied by the depression of the upper edge of the vertebral body, which gives the surgeon more room to repair the vertebral body by reducing the depressed endplate. However, it appears from the results of this study that such extensive injuries tend to cause accelerated IDD, leading to severe loss of IVD height and ICA in the future and even accompanying long‐term back pain symptoms in patients.^10^

### Limitations and Strengths

The advantages of this study are that (i) the classification of endplate injury morphology is simplified for the first time, which makes the classification system more suitable for rapid clinical prognosis analysis; (ii) the regularity between the morphology of endplate injury and the long‐term healing morphology of endplate was revealed for the first time, which can provide reference for future related research.

Some limitations must be pointed out. First, our study was limited to comparing objective indices of longer‐term postoperative such as changes in height and angle, and subjective indices related to patients' long‐term quality of life were not evaluated. Second, our average follow‐up time is about 20 months, and some of the parameters did not change significantly enough. We expect to show more differences between the different endplate injury types at longer term follow‐up. Third, the retrospective study design made it impossible to avoid selection bias. Fourth, this is a single‐center study with a small sample size. Therefore, the results of this study need to be confirmed by future multicenter, large sample size prospective studies.

## Conclusion

We reintegrated the endplate injury morphology and demonstrated the similarities and differences between the different endplate injury morphologies. We conclude that the fissure‐type injury endplate, although suggesting less damage in CT and MRI, shows significant loss of height and angle and other related indicators in the long‐term transition, with significant differences compared with other injury morphologies. This reminds clinicians that not only the area of the endplate injury affects the prognosis of patients with thoracolumbar fractures, but also the injury morphology of the endplate.

## Author Contributions

Qiuyu Yu and Hui Kang designed the study protocol. Yaozheng Han, Lintao Su, Zhongyi Guo, Changyu Lei and Jun Ma collected and analyzed the clinical data of patients. Qiuyu Yu and Kang Chen wrote the first draft of the manuscript together. Hui Kang, Jun Ma, and Kang Chen provided revision for intellectual content and final approval. The authors read and approved the final manuscript of the manuscript.

## Ethics Statement

This study was conducted according to the Helsinki Declaration and was approved by the Ethics Committee of the General Hospital of Central Theater Command of PLA (Ethics No. [2021] 059–01). Written informed consent was obtained for all participants.

## Funding Information

This work was supported by the Medical Youth Top Talent Project of Hubei Province (No.[2019]48#), Hubei Province 2023‐2024 Annual Health and Health Science Research Project (No.[2022]40#) (WJ2023M091), and Postdoctoral Fund of General Hospital of Central Theater Command (BSH018).

## Conflict of Interest Statement

The authors declare no potential conflicts of interest with respect to the research, authorship, and/or publication of this article.
